# Practical Protocols for Efficient Sampling of Kinase-Inhibitor Binding Pathways Using Two-Dimensional Replica-Exchange Molecular Dynamics

**DOI:** 10.3389/fmolb.2022.878830

**Published:** 2022-04-29

**Authors:** Ai Shinobu, Suyong Re, Yuji Sugita

**Affiliations:** ^1^ RIKEN Center for Biosystems Dynamics Research, Kobe, Japan; ^2^ Artificial Intelligence Center for Health and Biomedical Research, National Institutes of Biomedical Innovation, Health, and Nutrition, Ibaraki, Japan; ^3^ Theoretical Molecular Science Laboratory, RIKEN Cluster for Pioneering Research, Saitama, Japan; ^4^ RIKEN Center for Computational Science, Kobe, Japan

**Keywords:** molecular dynamics simulations, multi-dimensional replica-exchange simulations, generalized replica exchange with solute tempering, replica-exchange umbrella sampling, kinase-inhibitor binding

## Abstract

Molecular dynamics (MD) simulations are increasingly used to study various biological processes such as protein folding, conformational changes, and ligand binding. These processes generally involve slow dynamics that occur on the millisecond or longer timescale, which are difficult to simulate by conventional atomistic MD. Recently, we applied a two-dimensional (2D) replica-exchange MD (REMD) method, which combines the generalized replica exchange with solute tempering (gREST) with the replica-exchange umbrella sampling (REUS) in kinase-inhibitor binding simulations, and successfully observed multiple ligand binding/unbinding events. To efficiently apply the gREST/REUS method to other kinase-inhibitor systems, we establish modified, practical protocols with non-trivial simulation parameter tuning. The current gREST/REUS simulation protocols are tested for three kinase-inhibitor systems: c-Src kinase with PP1, c-Src kinase with Dasatinib, and c-Abl kinase with Imatinib. We optimized the definition of kinase-ligand distance as a collective variable (CV), the solute temperatures in gREST, and replica distributions and umbrella forces in the REUS simulations. Also, the initial structures of each replica in the 2D replica space were prepared carefully by pulling each ligand from and toward the protein binding sites for keeping stable kinase conformations. These optimizations were carried out individually in multiple short MD simulations. The current gREST/REUS simulation protocol ensures good random walks in 2D replica spaces, which are required for enhanced sampling of inhibitor dynamics around a target kinase.

## 1 Introduction

Ligand binding to a target protein or enzyme plays important roles in many biological processes which regulate protein functional activity (Du et al., 2016). Understanding of the binding processes directly contributes to the design of effective drugs which specifically bind to target proteins. Recently, the drug residence time on a protein has been attracting attention in the development of effective drugs (Bernetti et al., 2017; Schuetz et al., 2017). For this purpose, understanding the molecular mechanisms underlying protein-ligand binding processes, namely, binding pathways, transition states, encounter complexes, and binding kinetics, are essential, as well as sampling stable ligand-bound structures. Unlike most stable bound poses, transient and dynamic information is hardly accessible by experiments.

Molecular dynamics (MD) simulations are widely used to investigate conformational dynamics of biomolecules at the atomic level and are applied to many biological processes including protein-ligand binding/unbinding (De Vivo et al., 2016; Dickson et al., 2017; Bruce et al., 2018; Zhang et al., 2022). All-atom MD simulations can easily simulate protein dynamics on the 1–10 ms timescales, while high-performance MD-specialized computers are necessary to explore 1 ms or slower dynamics (Dror et al., 2011; Shan et al., 2011). Thus, conventional MD simulations of a protein-ligand complex are not sufficient for observing multiple binding/unbinding events, which are necessary for obtaining converged thermodynamics or free-energy landscapes. To go beyond, parallel trajectory MD methods (Silva et al., 2011; Plattner and Noé, 2015; Dickson, 2018; Tran et al., 2020) perform multiple short simulations and provide us with large amount of structural data for predicting long timescale dynamics. Another approach is the use of enhanced sampling MD methods such as replica-exchange MD (Sugita and Okamoto, 1999), metadynamics (Valsson et al., 2016), and others (Meng et al., 2015; Miao and McCammon, 2017; Spitaleri et al., 2018; Gobbo et al., 2019; Hénin et al., 2022) to explore a wider conformational space of systems with rugged energy landscapes by overcoming high energy barriers between multiple minimum states. Replica-exchange MD (REMD) (Sugita and Okamoto, 1999; Sugita et al., 2000) effectively overcome energy barriers through the exchange of system parameters between independently running replicas. In temperature REMD, high temperature replicas sample various conformations including unfolded, extended, or other flexible ones, while low temperature replicas explore stable structures existing at different energy minima through the replica exchange. In replica exchange with solute tempering (REST REST/REST2) (Liu et al., 2005; Terakawa et al., 2011; Wang et al., 2011), a specific region of interest is selected as “solute,” and “solute temperature” exchanges are attempted with a reduced number of replicas. Hence, REST/REST2 is applicable to larger biological systems than temperature-REMD owing to the reduced computational cost. Recently, we generalized the definition of “solute” in REST2 by selecting a part of the molecule of interest and/or a part of the potential energy function terms as “solute”. This method, which we refer to as the generalized REST (gREST) (Kamiya and Sugita, 2018), can reduce the number of replicas even further while observing efficient conformational dynamics of proteins or protein-ligand complexes. For instance, in gREST simulations of protein-ligand binding, the solute is defined as a target ligand as well as amino-acid sidechains near the target protein binding site, which accelerates ligand dynamics more than in REST2 simulations, where only the ligand molecule is selected as “solute”. The gREST method was applied for the prediction of the correct binding pose (Niitsu et al., 2019) and affinities, when combined with absolute binding free energy calculations (Oshima et al., 2020). The replica-exchange umbrella sampling (REUS) method (Sugita et al., 2000; Fukunishi et al., 2002) exchanges geometrical parameters along a predefined collective variable (CV). This method is also applicable to large biological systems, if a good CV is used for describing the target conformational motion.

It is noteworthy that different parameters can be exchanged in a multidimensional fashion to further enhance conformational sampling of various biological systems (Sugita et al., 2000). Multidimensional REMD was first applied in protein-ligand binding simulations by Kokubo et al.(2013) where they combined REST2 with REUS (the REST2/REUS method). In their study, a target ligand was selected as solute in REST2 and the protein-ligand distance was used as a CV in REUS. After the success of this approach, we replaced REST2 with gREST and applied the gREST/REUS method to inhibitor binding/unbinding in the c-Src kinase/PP1 complex (gREST/REUS) (Re et al., 2019). We briefly describe the gREST/REUS method in the Supplementary Text and Figure S1. The simulations could enhance inhibitor dynamics around c-Src kinase and we observed a total of about 100 binding/unbinding events for all replicas. Using the well-converged free-energy landscapes of protein-ligand binding processes, multiple binding pathways, transition states, encounter complex structures, and other atomistic insights were obtained for the c-Src kinase-PP1 complex in solution.

**FIGURE 1:**
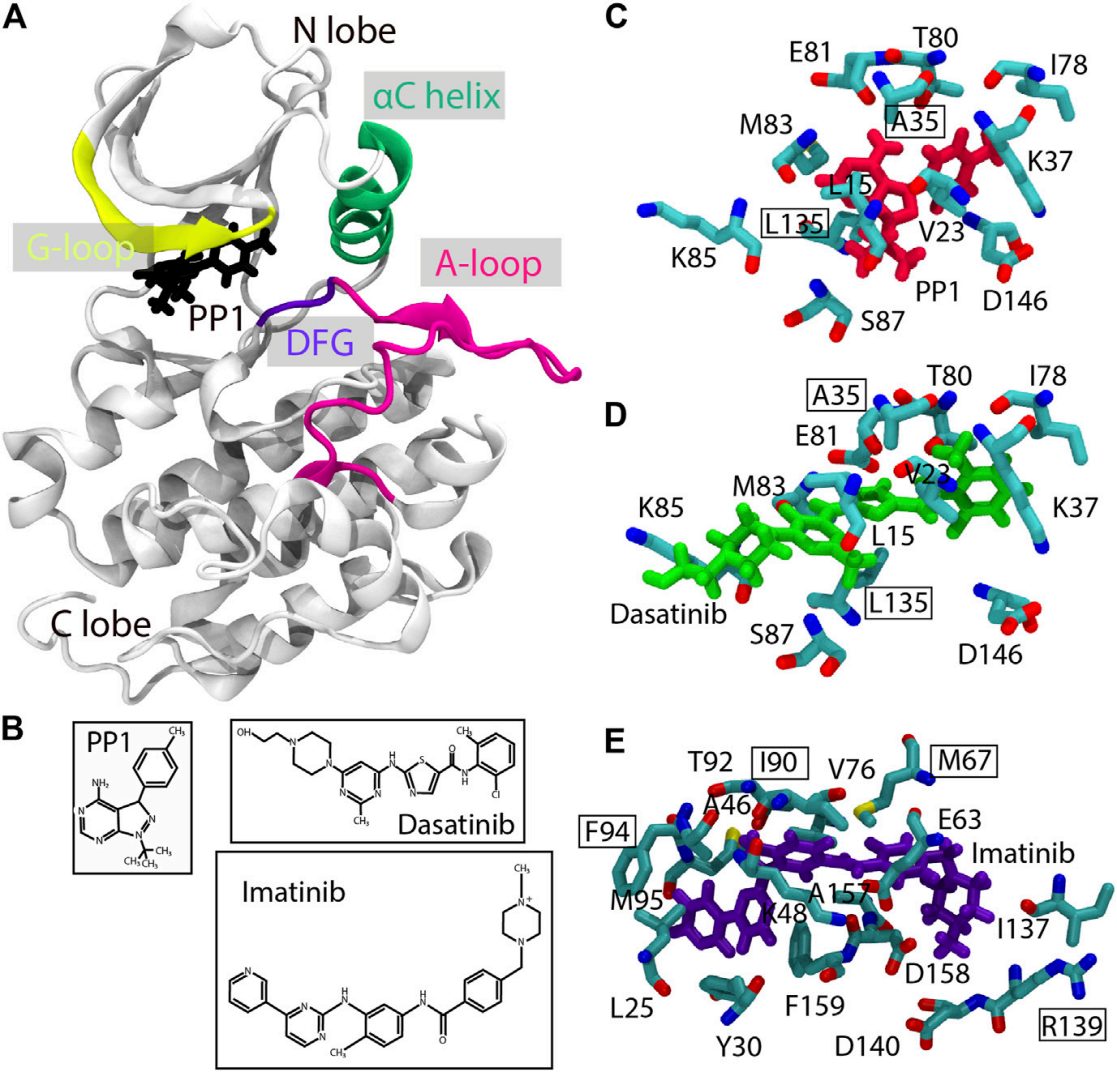
Structures of the Src-PP1, Src-Dasatinib, and Abl-Imatinib complexes. **(A)** Src-PP1 model from X-ray structures (PDB ID: 1Y57/1QCF). **(B)** chemical structures of PP1, Dasatinib, and Imatinib. **(C)**–**(E)** Binding site of Src-PP1 **(C)**, Src-Dasatinib **(D)** and Abl-Imatinib **(E)** from X-ray structures (PDB ID: 1Y57/1QCF, 1Y57/3G5D and 1IEP/2OIQ for protein/ligand, respectively). PP1, Dasatinib, and Imatinib are colored red, green, and purple, respectively. Protein residues used as gREST solute regions are also shown. Residues used as protein COM for REUS CV are outlined.

The gREST/REUS method is theoretically applicable to any biological system for studying molecular mechanisms of protein-ligand binding/unbinding processes. However, the size and flexibilty of the ligand increase the computational difficulty. Here, we re-examine the practical protocols of the two-dimensional (2D) gREST/REUS protein-ligand binding simulations and apply them for three kinase-inhibitor systems: c-Src kinase with PP1 (Src-PP1), c-Src kinase with Dasatinib (Src-Dasatinib), and c-Abl kinase with Imatinib (Abl-Imatinib) (Figure 1). As the size and flexibility of the ligand increases in the aforementioned order, binding simulations are expected to be more challenging. Kinase-inhibitor binding processes have been subjected to both long-time conventional MD (Shan et al., 2011; Morando et al., 2016; Paul et al., 2020; Sohraby et al., 2020) and enhanced sampling MD simulations (Yang et al., 2009; Lin et al., 2013; Tiwary et al., 2017; Gobbo et al., 2019; Koneru et al., 2019; Narayan et al., 2020; Spitaleri et al., 2020; Narayan et al., 2021; Shekhar et al., 2021). However, to gain more atomistic insight on protein-ligand binding processes, better computational algorithms and practical protocols are necessary. In this paper, we describe how to optimize parameters and procedures for the setup of gREST/REUS simulations and target biomolecular systems. The role of flexible inhibitor binding in c-Src/c-Abl kinases will be discussed in a separate paper, thus here we focus on the practical issues and the protocols, which are non-trivial when performing gREST/REUS simulations with more than a hundred replicas. The protocols presented here can be useful for carrying out ligand binding/unbinding simulations of various biomolecular systems with the gREST/REUS method on massively parallel supercomputers or GPU clusters.

## 2 Methods

### 2.1 The gREST/REUS Simulation Protocols

The 2D-REMD methods such as gREST/REUS typically require a large number of replicas (i.e., more than 100 replicas), while they can realize better random walks in replica space including the bound, intermediate, and unbound states of the protein/ligand complexes. The preparation of replicas and the choice of solute temperatures in gREST and/or collective variables in REUS directly affects the conformational sampling efficiency. For instance, if there exist large distribution gaps between replicas, we cannot observe good random walks in replica space. This situation is equivalent to performing multiple independent REMD simulations with smaller number of replicas, which might lead to missing important intermediate structures and slow convergences of thermodynamic data. Initial setups of the gREST/REUS simulations are thus, essential for successful gREST/REUS calculations and for obtaining reliable simulation results.

In gREST/REUS, replica random walks are necessary in both the gREST and REUS dimensions. The former is realized only when the solute region and replica temperatures are defined appropriately, and we can observe sufficient overlaps in potential energies between replicas at neighboring solute temperatures. In REUS simulations, the choice of CVs, replica distributions along the CV, and proper force constants in US potentials are all important. There are many parameters and choices of procedures in gREST/REUS simulations with more than 100 replicas. For simplifying the parameter optimization, we tuned the parameters in each dimension separately using multiple short MD or gREST/REUS simulations, as described below.

#### 2.1.1 Definition of the Protein-Ligand Distance as a CV for REUS

The protein-ligand distance is commonly used in binding MD simulation studies. The distance is usually measured as that between the centers of mass (COMs) of the backbone atoms of the selected binding site residues (protein anchor sites) and ligand heavy atoms (ligand COM). For Src-PP1 and Src-Dasatinib, the backbone atoms of Ala35 and Leu135 in c-Src kinase are used as the protein anchor site. All the heavy atoms in PP1 and Dasatinib were used for obtaining the ligand COM, since they are small compounds with less conformational flexibilities than Imatinib, which is composed of five rings. There are multiple choices for Abl-Imatinib for the protein anchor sites and the ligand COM. As the former, we tested four choices: “2 sites” (Ile90 and Arg139), “3 sites” (Ile90, Arg139, and Phe94), “4 sites” (Ile90, Arg139, Phe94, and Met67), and “5 sites” (Ile90, Arg139, Phe94, Met67, and Phe159), respectively. For the ligand COM, four definitions including a single ring (“Ring3”), three rings (“Ring135” and “Ring 234”), and all rings (“Ring all”) were examined. We expect that Imatinib flexibility is important not only near the binding site but also in the intermediate or unbound structures. A good combination of the protein anchor sites and the ligand COM may reduce the number of possible protein-ligand complex structures near the binding sites. In our protocols, the ligand COM definition was first examined for Imatinib and then, multiple choices of the protein anchor sites were tested for Abl-Imatinib simulations.

#### 2.1.2 Preparation of Initial Structures in REUS

Thirty replicas were used for covering the protein-ligand distance in the range of 3–18 Å for Src-PP1, and 3–23 Å for Src-Dasatinib and Abl-Imatinib. We obtained the initial structure of each replica using two US simulations: In the “forward pull” simulation, the ligand was gradually pulled away from the protein binding site, while it was subsequently pulled back to the bound pose in the “reverse pull” simulation. Each replica was simulated for 300 ps with a force constant of 4 kcal/mol/Å^2^. Positional restraints on the protein Cα atoms with a force constant of 1 kcal/mol/Å^2^ were necessary during the pulling simulations to prevent artificial deformations of the protein. In this stage, the 30 initial structures were set in equidistance in the REUS dimension.

#### 2.1.3 Tuning of Solute Temperatures in gREST

The solute region in gREST was defined as the dihedral angle and the nonbonded energy terms of the ligands and binding-site residues of the proteins (ca. 10 residues defined as SITE residues in the X-ray structure as shown in Figures 1C–E, and listed in Supplementary Table S1). We determined the solute temperatures using the automatic parameter tuning tool in the GENESIS MD program (Kobayashi et al., 2017). Given initial temperatures and desired acceptance ratio as inputs, the tool finds a set of solute temperatures which satisfies the desired acceptance ratio. The initial temperatures and the target acceptance ratio were set in the range of 310–663 K and 0.2, respectively. We performed five rounds of the tuning simulations (1.1 ns for each replica), by gradually increasing the frequency of exchange attempts (from every 0.21 ps in the first round to every 2.1 ps for final round), until temperature values were converged. The tuning was performed in 1D-gREST simulations at the bound (protein-ligand distance of 3.0 Å), intermediate (10.3 Å for Src-PP1, and 15.0 Å for Src-Dasatinib and Abl-Imatinib), and the unbound states (18.1 Å for Src-PP1, and 23 Å for Src-Dasatinib and Abl-Imatinib). The final temperature values were taken as the average of those obtained at the above three states.

#### 2.1.4 Determination of REUS Parameters

To ensure sufficient potential energy overlaps between adjacent replicas, which is a pre-requisite for good REUS performance, we conducted several short trial simulations, while manually tweaking the location and force constants. At each round, we assessed the distribution overlaps between replicas and the acceptance ratios, and accordingly modified the REUS parameters, namely, the center position and the force constant of each harmonic umbrella potential. The tuning procedures were repeated in 1D-REUS simulations at three solute temperatures: 310 K (at lowest) for all three systems, 478 K, 471 K, and 440 K (at middle), and 692 K, 663 K, 590 K (at highest) for Src-PP1, Src-Dasatinib, and Abl-Imatinib, respectively.

### 2.2 System Preparation

The initial structure of Src-PP1 was taken from our previous work (Re et al., 2019). In brief, we extracted the kinase domain (residues 260–533, renumbered 2–275 in this work) from the X-ray crystal structure of the active-like c-Src kinase (PDB ID: 1Y57) (Cowan-Jacob et al., 2005) and replaced the co-crystallized ligand with PP1 bound to c-Src (PDB ID: 1QCF) (Schindler et al., 1999). The initial structures of Src-Dasatinib and Abl-Imatinib were constructed with the same modeling protocol used for Src-PP1. For Src-Dasatinib, we used the kinase domain of c-Src kinase (PDB ID: 1Y57) (Cowan-Jacob et al., 2005) and replaced the co-crystallized ligand with Dasatinib from an X-ray structure (PDB ID: 3G5D) (Getlik et al., 2009). Similarly, for Abl-Imatinib, we used the kinase domain (residues 225–498, renumbered 2–275 in this work) of c-Abl kinase (PDB ID: 1IEP) (Nagar et al., 2002) and the ligand structure from an X-ray structure (PDB ID: 2OIQ) (Seeliger et al., 2007). Each kinase-inhibitor complex was solvated with water molecules, where the number of water molecules was 7,698, 13,992, and 17,485 for Src-PP1, Src-Dasatinib, and Abl-Imatinib, respectively. The size of the simulation boxes for Src-Dasatinib and Abl-Imatinib was larger than for Src-PP1 because the farthest REUS replica (created by the US pulling simulations) was placed farther from the binding site (23 Å vs 18Å). The systems were neutralized by adding sodium counter ions (six for Src-PP1 and Src-Dasatinib and eight for Abl-Imatinib). Each system was minimized for 1,000 steps while applying a positional restraint of 10.0 kcal/mol/Å^2^ on protein backbone atoms. Then it was gradually heated to 310 K in the NVT ensemble for 105 ps, followed by equilibration in the NPT ensemble for 105 ps. Finally, the system was equilibrated for 1.05 ns in the NPT ensemble without restraining the protein atoms. Modeling was performed using AmberTools16 (Case et al., 2021).

### 2.3 MD Simulation

Simulations were performed using the GENESIS MD program (Jung et al., 2015; Kobayashi et al., 2017) version 2.0 beta (Jung et al., 2021). The AMBER ff99SB-ILDN (Hornak et al., 2006; Lindorff-Larsen et al., 2010) force field was used for the proteins, GAFF (Wang et al., 2004) (with AM1-BCC) for the ligands, and the TIP3P (Jorgensen et al., 1983) was used for water molecules. Bonds involving hydrogen atoms were constrained using the SHAKE algorithm (Ryckaert et al., 1977). Water molecules were kept rigid using the SETTLE algorithm (Miyamoto and Kollman, 1992). Long-range electrostatic interactions were evaluated using the Particle-mesh Ewald summation (Darden et al., 1993; Essmann et al., 1995). The cutoff distance for non-bonded interaction was 8 Å. The NVT ensemble was used with the Bussi thermostat (Bussi et al., 2007) for keeping the temperature at 310 K, with a temperature coupling time of 5 ps. A timestep of 3.5 fs was used with the RESPA integrator (Tuckerman et al., 1992) and hydrogen mass repartitioning (HMR) (Feenstra et al., 1999) was applied on solute atoms with an HMR ratio of 3.0 (Jung et al., 2021).

Eight gREST replicas and 30 REUS replicas were used in the 2D-gREST/REUS simulation. In total, the number of replicas in each run was 240. All replicas were equilibrated for 1.05 ns without exchange attempts, followed by production runs. Exchanges were attempted every 2.1 ps alternatively in the gREST and the REUS dimensions. The gREST/REUS simulations were performed for 500 ns per replica for Src-PP1, 750 ns per replica for Src-Dasatinib, and 1,000 ns per replica for Abl-imatinib. For Src-PP1, two simulations using initial replicas from either the “forward pull” or “reverse pull” US simulations were performed (referred to as Src-PP1 and Src-PP1-Rev, respectively). All simulations steps (except the US pulling simulations for creating the initial REUS replicas) were performed without any restraints on protein atoms. The total simulation time in the current work was 660 µs. Frames for analysis were written every 10.5 ps. Simulations were performed on the supercomputer Fugaku^1^ using 480 nodes. The GENESIS 2.0 beta version was optimized to run on Fugaku and obtained a speed of >50 ns/day. The details of the systems and the simulation conditions are summarized in Table 1.

**TABLE 1:** System models and simulation details.

System	Src-PP1 (Forward/Reverse^a^)	Src-dasatinib	Abl-imatinib
Protein structure	1Y57 Cowan-Jacob et al. (2005)	1Y57 Cowan-Jacob et al. (2005)	1IEP Nagar et al. (2002)
Ligand structure	1QCF Schindler et al. (1999)	3G5D Getlik et al. (2009)	2OIQ Seeliger et al. (2007)
Number of atoms	27,549 (7,698 waters)	46,240 (13,992)	56,952 (17,485)
gREST solute temperature range, K	310–692	310–663	310–590
REUS distance range, Å	3.0–17.9/3.0–18.05^b^	3.0–23.1	2.7–23.0
Simulation time per replica, ns	500	750	1,000

a For simulations that were initiated from REUS, replicas obtained from the forward and reverse simulations.

b Range of values for forward simulations/range of values for reverse simulations.

## 3 Results

### 3.1 Tuning the Definition of Protein-Ligand Distance in the REUS Dimension

**FIGURE 2:**
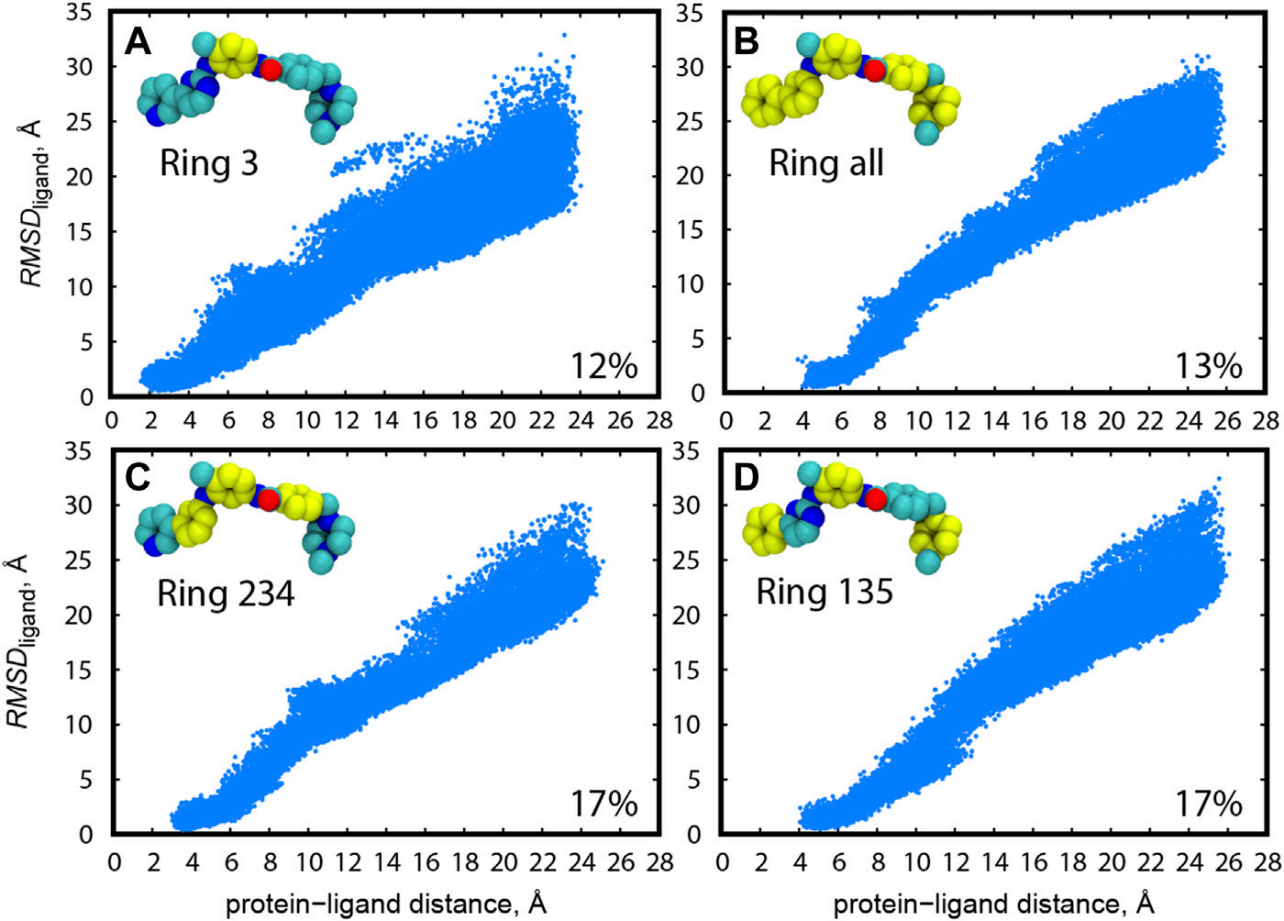
Distribution of *RMSD*_ligand_ along the protein-ligand distance for trial simulations (10 ns) for all replicas (1–240) of Abl-Imatinib for different definition of ligand COM atoms: **(A)** “Ring 3”, **(B)** “Ring all”, **(C)** “Ring 234”, and **(D)** “Ring 135”. Atoms used for ligand COM definitions are colored yellow. Ligand rings are numbered from 1 to 5, starting from the left. Protein atoms used for COM are backbone atoms of I90 and R139. The percentage of replicas that reached the bound pose is written on the bottom right of each panel.

As for Src-PP1 and Src-Dasatinib, we defined the protein-ligand distance using the “2 site” model (Ala 35 and Leu135) in c-Src kinase for the protein anchor sites and all the heavy atoms for calculating the ligand COM. Due to the inhibitor size and flexibility, we tested multiple choices of the protein anchor sites and the ligand COMs for Abl-Imatinib by short (10 ns) gREST/REUS trial simulations. Figure 2 shows the distribution of the ligand RMSD (*RMSD*_ligand_) with respect to the bound pose of the X-ray crystal structure (2OIQ) (Seeliger et al., 2007) along the protein-ligand distance. As for the ligand COM, three rings (“Ring 135” and “Ring 234”), a single ring (“Ring 3”) and all rings (“Ring all”) were tested when we used “2 site” (Ile90 and Arg139) as the protein anchor site in c-Abl kinase. The probability of finding the bound pose, which we defined as the percentage of replicas that reached *RMSD*_ligand_ < 1 Å at least once during the simulation, is also shown. An efficient pose sampling should give a linear correlation with narrow distribution. CVs with multiple rings in Imatinib (“Ring all”, “Ring 234”, and “Ring 135”, Figures 2B–D) display linear and narrow distributions overall, compared to the single ring (“Ring 3”, Figure 2A). The latter could possess various conformations at the same distance, likely worsening the efficiency. “Ring 234” (Figure 2C) and “Ring 135” (Figure 2D) both have higher probabilities of finding the bound pose, while the latter shows slightly narrower distribution in the range of short protein-ligand distances. These results suggest that three anchor sites (a molecular center and both edges, “Ring 135”) is the practical choice for Abl-Imatinib.

**FIGURE 3:**
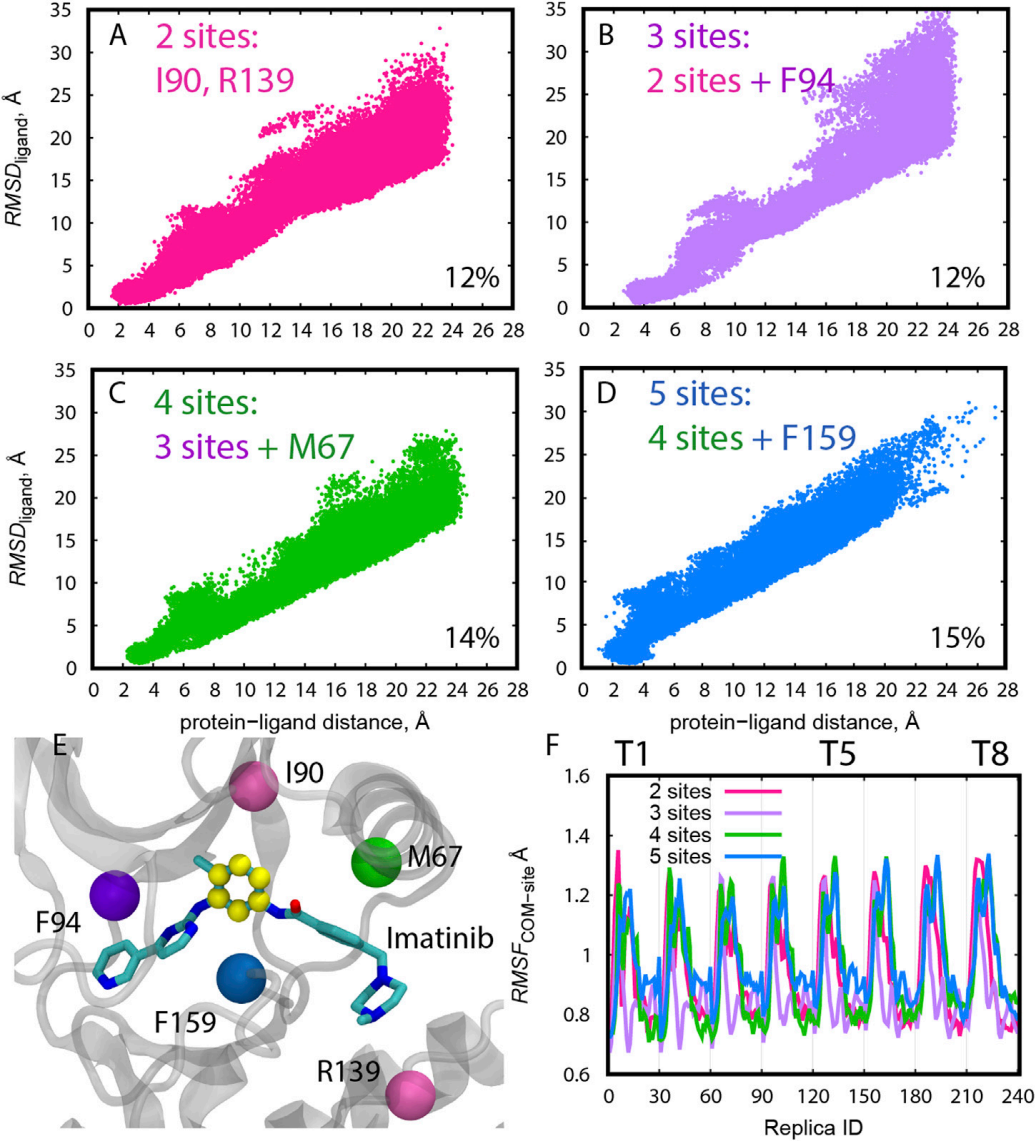
**(A–D)** Distribution of *RMSD*_ligand_ along the protein-ligand distance for trial simulations (10 ns) for all replicas (1–240) of Abl-Imatinib for different definition of protein COM atoms. The percentage of replicas that reached the bound pose is written on the bottom right of each panel. **(E)** Definition of COM atoms. Cα atoms of the residues used for COM definition of the protein are shown as colored balls. Atoms used for ligand COM definition (“Ring3”) are colored yellow. **(F)** Root-mean-square-fluctuation (RMSF) of the COM of the protein anchor site atoms calculated for the 10 ns trial simulations. The reference structure used for calculating the RMSF was the initial X-ray structure. For the purpose of RMSF calculations replicas were sorted according to their REUS and gREST parameters as follows. Each group of 30 replicas belong to a single solute temperature, where replicas 1–30 represent T1 (lowest temperature), and replicas 211–240 represent T8 (highest temperature). Within each temperature, replicas are ordered according to increasing protein-ligand distances such that replicas 1 and 30 represent the smallest and largest distances, respectively.

At the same time, we tested four choices for the protein anchor sites using “Ring 3” as the ligand COM in Abl-Imatinib simulations (Figure 3). The overall distribution of *RMSD*_ligand_ becomes narrow with increasing number of protein anchor sites. The probability of finding the bound pose is higher for “4 sites” and “5 sites” (14–15%, Figures 3C,D) than “2 sites” and “3 sites” (12%, Figures 3A,B), suggesting that two or three anchor sites are not sufficient to resolve the bound conformations of a ligand as large as Imatinib. “5 sites” produces a relatively wide distribution compared to “4 sites” at the bound region (∼4 Å, Figure 3D). Increasing the number of residues in the protein anchor sites (Figure 3E) is effective for resolving the ligand position and orientation but bears the risk of making protein anchor sites unstable. The COMs with “5 sites” indeed fluctuated more than the others through the replicas (Figure 3F). Consequently, the “4 sites–Ring135” pair was chosen as the best combination for Abl-Imatinib.

### 3.2 Preparation of Initial Structures in REUS From the Pulling Simulations

**FIGURE 4:**
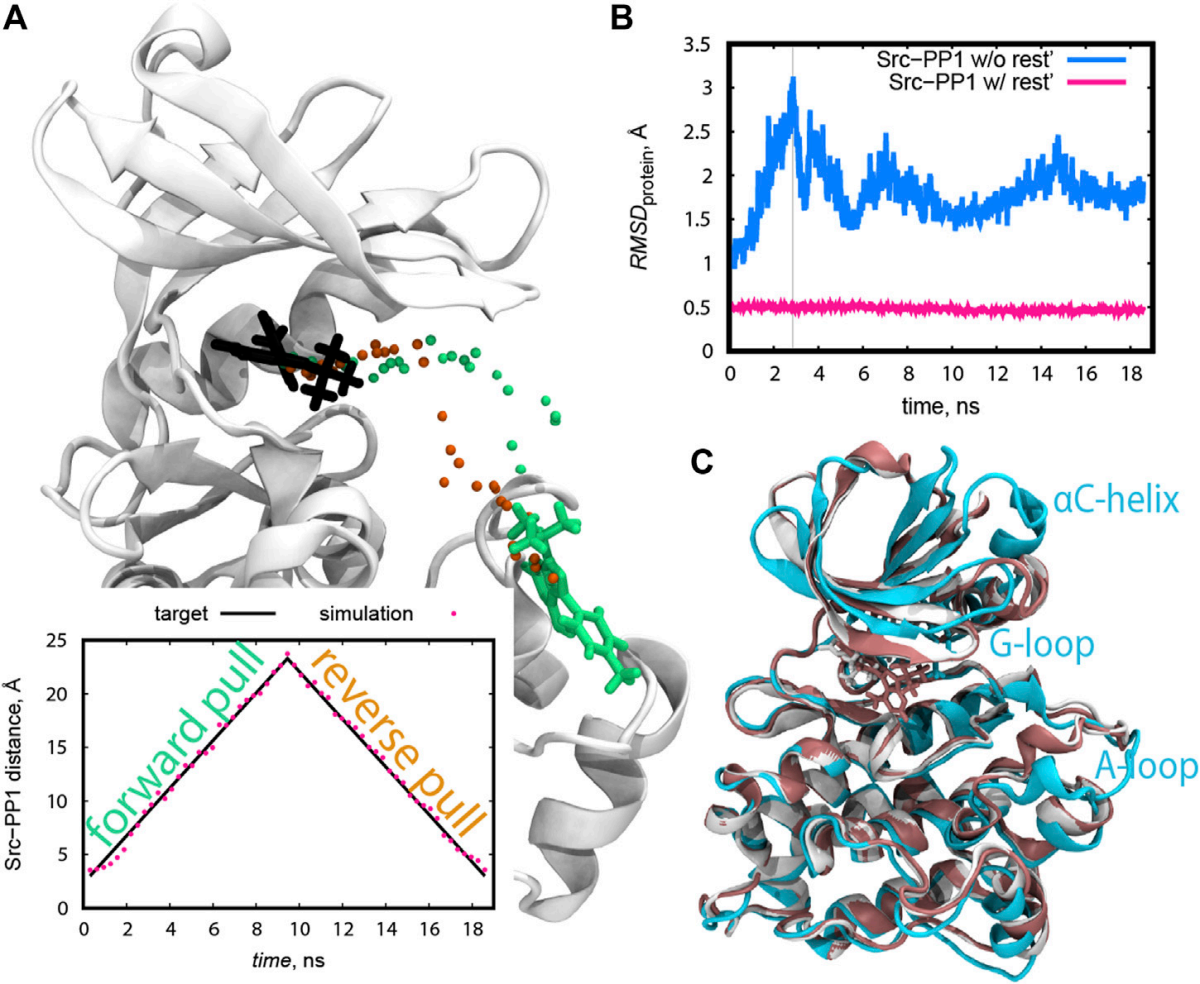
**(A)** The course of the ligand PP1 during the pulling simulations represented as the coordinates of the C9 atom of PP1 at the end of each pulling step. The forward and reverse pulling directions are represented by green and orange dots, respectively. The locations of the ligand in the X-ray structure and in the last forward pulling simulation are shown in licorice representation in black and green, respectively. Inline plot: Src-PP1 distance for pulling simulations using a force constant of 4 kcal/mol/Å^2^. Target distances are shown as black lines. **(B)** RMSD of protein backbone atoms during pulling simulations of PP1 from c-Src kinase with and without 1 kcal/mol/Å^2^ positional restraints on the protein Cα atoms. The pulling was performed by applying the force of 4 kcal/mol/Å^2^ over the protein-ligand COM distance. The *X*-axis represents the total time of concatenated consecutive pulling simulations. **(C)** Snapshots of the protein from the simulations described in **(B)** with (pink) and without (cyan) positional restraint on protein atoms. The snapshots were taken at the time marked by the grey vertical line in **(B)**.

The initial structures along the protein-ligand distance CV were prepared from the following pulling simulations. Both forward (pulling away from the bound pose) and reverse directions (pulling back to the bound pose) were examined in the case of Src-PP1. The resulting initial pathways differed from each other (Figure 4A), suggesting that “dual direction pulling” could reduce the initial structure dependence and improve the convergence of the simulation results. To prepare the initial coordinates at each of the desired protein-ligand distances along the path in short simulations (9 ns per each), a rather strong force constant (4 kcal/mol/Å^2^) of the umbrella potential was required (Figure 4A). Note that the pulling simulations can introduce an artificial structure change in the protein. In the case of Src-PP1, the structures around the αC-helix, the G-loop, and the A-loop region significantly deviated from the X-ray crystal structure (Figures 4B,C). Since these regions directly relate to the binding mechanism, we also applied 1 kcal/mol/Å^2^ restraints on the protein Cα atoms to avoid the artificial structure changes.

### 3.3 Tuning of Solute Temperatures in gREST

**FIGURE 5:**
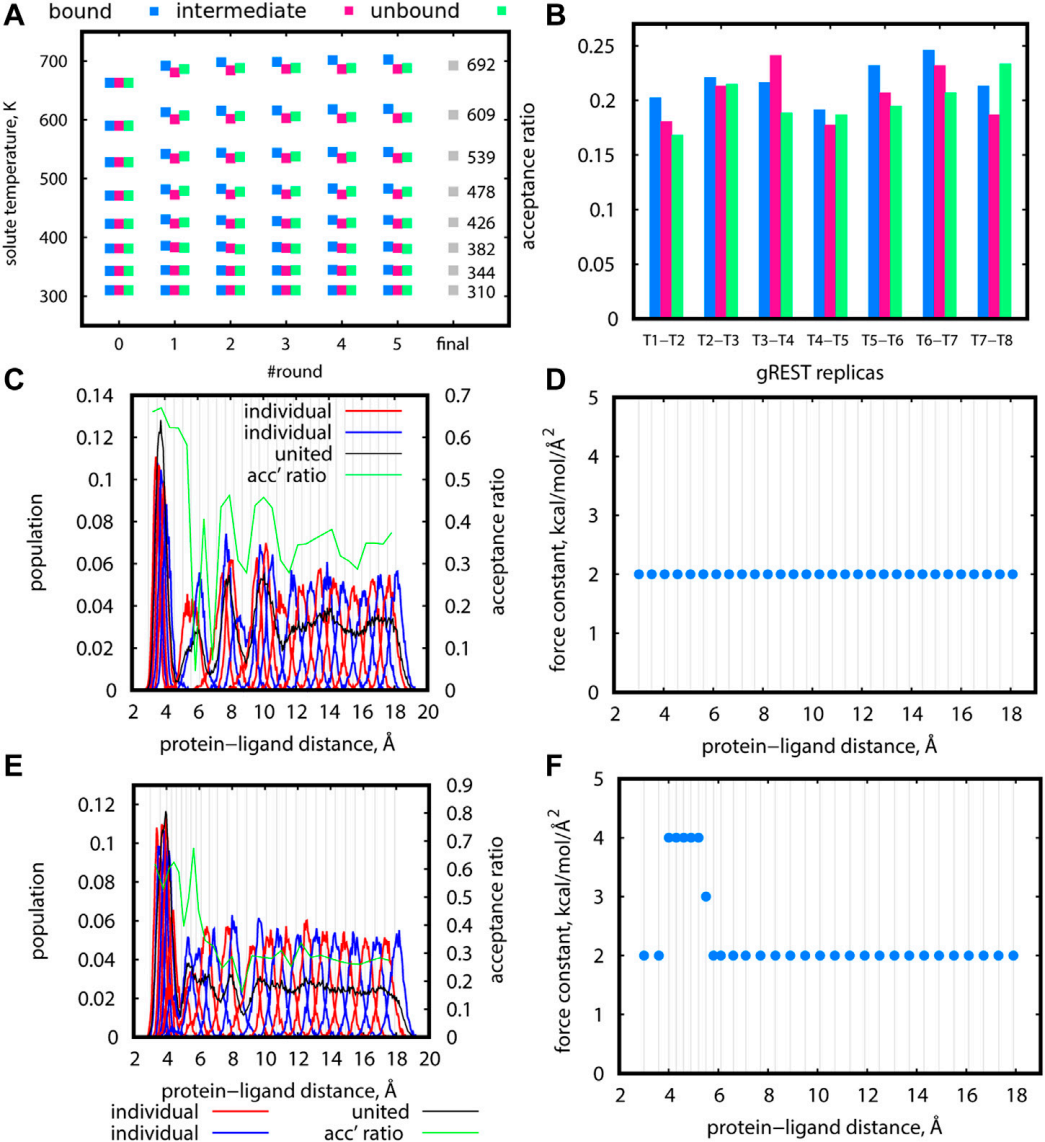
**(A)** Final gREST temperatures after each automatic tuning round at three protein-ligand distances for the Src-PP1 system with a target acceptance ratio of 0.20. Round “0” specifies the initially guessed temperatures. Round “final” is the final temperature obtained from averaging the final temperatures for the three distances. **(B)** Acceptance ratios between adjacent replicas in simulations using the temperatures obtained in round “5” of the gREST tuning procedure described in **(A)**, at three protein-ligand distances. **(C)**, **(E)** Distribution of replicas according to their REUS distance for short trial simulations of 5.3 ns at 310 K for Src-PP1 (using initial replicas from the forward pulling simulations). Distributions of adjacent individual replicas (“individual”) are shown in alternating red/blue lines for better visibility. Distributions of all replicas (“united”) are shown in black lines. Population values for “united” data were scaled to match the “individual” populations. Acceptance ratios between adjacent REUS replicas are shown in green lines. **(D)**, **(F)** Force constants used for the simulations **(C)** and **(E)**, respectively. Vertical lines mark the protein-ligand COM distance at each replica. Blue dots mark the value of the force constant used at each REUS distance.

Solute temperatures in gREST could be determined rather effortlessly using the automatic tuning tool in GENESIS (Kobayashi et al., 2017). For Src-PP1, we set the initial temperatures in the range of 310–663 K, which is much narrower than our previous work (Re et al., 2019). This change markedly improved the sampling along the solute temperature space. In addition, there are two key points in determining the temperatures. First, multiple rounds of tuning are desired. Figure 5A shows the solute temperatures determined at each of the five tuning rounds, where we set the final temperature at each round as the initial temperature for the subsequent round. The temperature values changed for the first few rounds and converged. Second, tuning at different protein-ligand distances are desired. For Src-PP1, we performed the tunings at protein-ligand distances of 3.0 Å (“bound”), 10.3 Å (“intermediate”), and 18.1 Å (“unbound”) distances. The resulting temperatures slightly differ in the three states (Figure 5A), and we therefore took their average at the final round to obtain the final set of solute temperatures. The resulting solute temperatures provided uniform acceptance ratios along the gREST replicas (Figure 5B). Temperature tuning for Src-Dasatinib and Abl-Imatinib was performed using the same scheme. In practice, we manually changed the value of target acceptance ratios at each round for obtaining the final acceptance ratio of 0.2. The final sets of solute temperatures are listed in Supplementary Table S1.

### 3.4 Tuning of the REUS Parameters

The tuning of distance replicas and force constants in REUS simulations was more challenging, and several trial rounds were required for obtaining proper values. For Src-PP1, we started with even-spaced distance replicas and a uniform force constant of 2 kcal/mol/Å^2^ at 310 K (Figures 5C,D). The sampled distance distribution was uneven. For example, the regions around 4 Å, 6 Å, and 9 Å are poorly covered, while there is an overlap in the region under 4 Å giving a large population in that region. The acceptance ratios around 6 Å drops to nearly zero, indicating almost no exchanges between replicas in that region. Accordingly, we put more replicas in poorly covered regions and set the force constants in those replicas to larger values (3 and 4 kcal/mol/Å^2^) (Figures 5E,F). This modification resulted in better coverage of the REUS space and acceptance ratios of above 0.2 for most replicas, ensuring the occurrence of replica exchanges throughout the REUS dimension. Nevertheless, we still observed an overly large population of replicas in the bound region of under 4 Å alongside regions with poor coverage. The gap cannot be eliminated altogether since poorly covered regions represent high energy regions on the free energy landscape. This demonstrates the necessity of performing replica exchanges in two dimensions where increasing the temperature will facilitate the crossing of high energy barriers. The final REUS replica placements and force constant values are given in Supplementary Table S1.

### 3.5 Sampling Efficiency of the gREST/REUS Simulations After Parameters Tuning

As production runs, gREST/REUS simulations with 240 replicas were executed on the three systems, Src-PP1 (500 ns), Src-Dasatinib (750 ns), and Abl-Imatinib (1,000 ns), using the optimal parameters determined as described in previous sections. In the following sections, we quantify their sampling efficiencies in replica space and in the conformational space of the kinase-inhibitor complexes.

#### 3.5.1 Random Walks in the gREST Dimension

**FIGURE 6:**
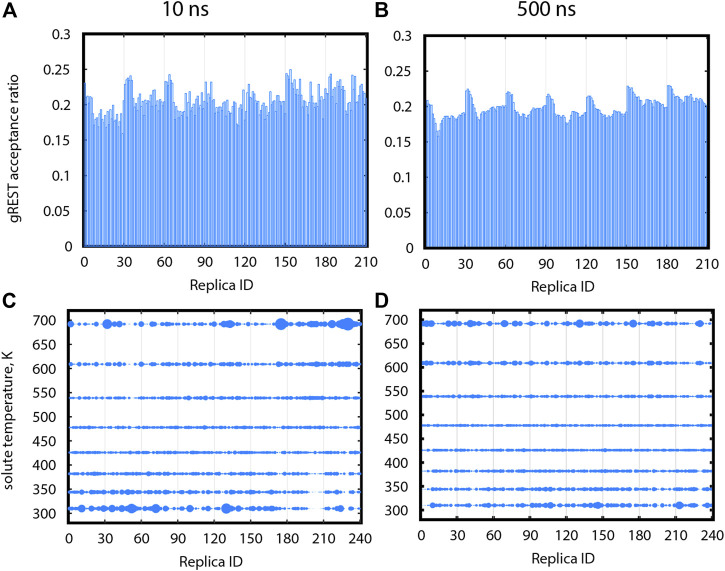
Sampling in gREST dimension after 10 ns **(A,C)** and after 500 ns **(B,D)** for gREST/REUS simulations of Src-PP1. **(A)**, **(B)** Acceptance ratios between each replica and the gREST replica adjacent and above it. **(C)**, **(D)** Relative population for each replica, at each gREST replica. Sphere size is proportional to the population. Replicas assigned different initial solute temperatures are separated by vertical lines, where replicas 1–30 were assigned the initial temperature of 310 K (T1), replicas 31–60 were assigned the initial temperature of T2, etc.

Proper exchanges in the gREST dimension will allow replicas to go back and forth between low and high solute temperatures to sample high energy conformations. Figures 6A,B show acceptance ratios between adjacent gREST replicas for Src-PP1 after 10 and 500 ns, respectively. Acceptance ratios average around 0.2 as early as 10 ns. Figures 6C,D show the relative population at each solute temperature visited by individual replicas. Ideally, a uniform sampling is desired, in which each replica visits each temperature evenly. In a short simulation time (10 ns), all solute temperatures were already visited, although the replicas preferred the lowest and highest temperatures. After 500 ns, the sampling becomes more uniform, where the excessive population at the lowest and highest temperatures for some of the replicas flattens out. We found similar trends in the other two systems (Supplementary Figure S2), while the convergence becomes slower with increasing ligand size from PP1 to Dasatinib to Imatinib.

#### 3.5.2 Random Walks in the REUS Dimension

**FIGURE 7:**
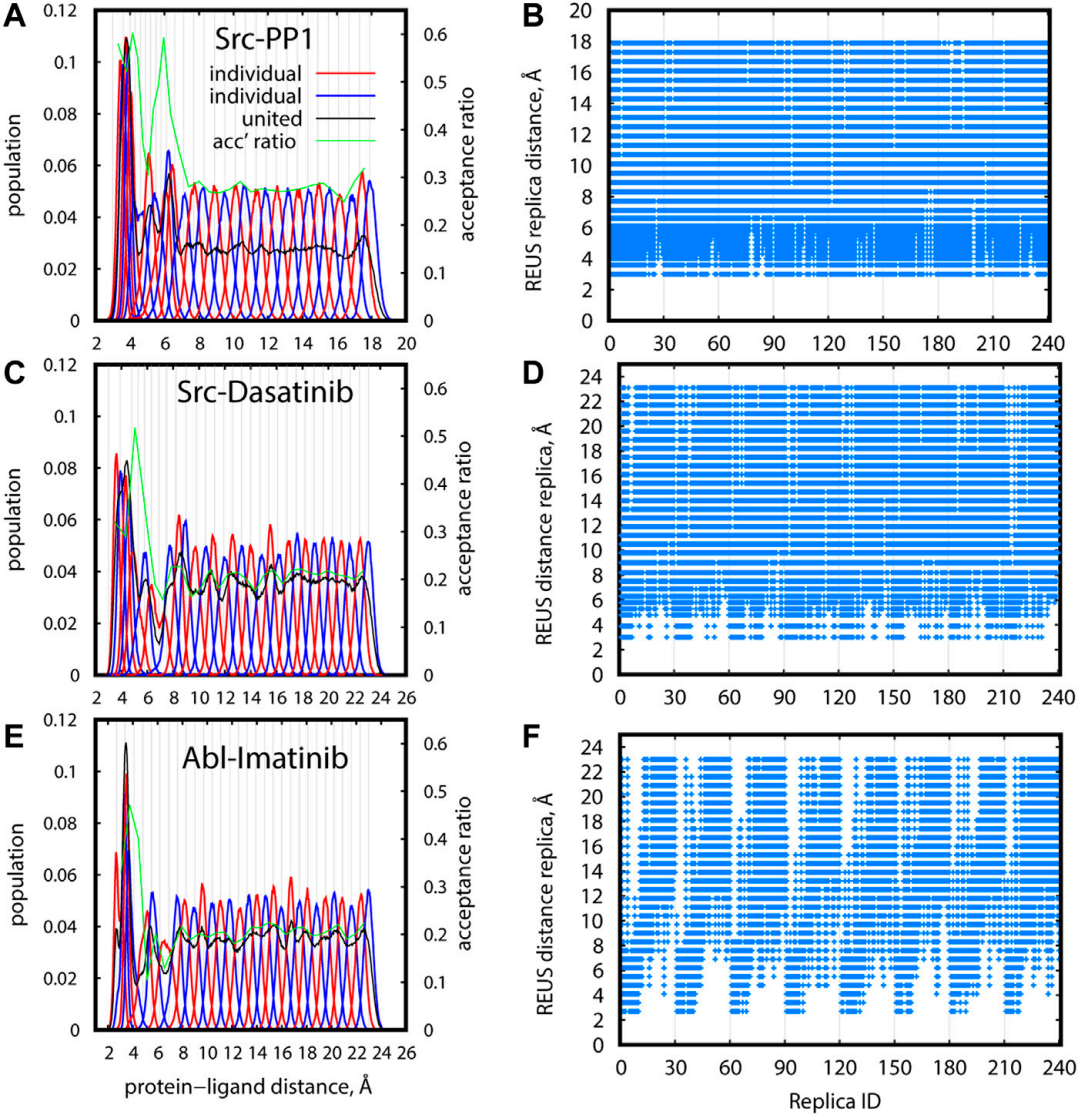
Efficiency of sampling in REUS space for gREST/REUS simulations at 310 K for 500-ns Src-PP1 **(A,B)** 750-ns Src-Dasatinib **(C,D)**, and 1,000-ns Abl-Imatinib **(E, F)**. **(A)**, **(C)**, **(E)** Distribution of replicas according to their REUS distance. Distributions of adjacent individual replicas (“individual”) are shown in alternating red/blue lines for better visibility. Distributions of all replicas (“united”) are shown in black lines. Population values for “united” data were scaled to match the “individual” populations. Acceptance ratios between adjacent REUS replicas are shown in green lines. **(B)**, **(D)**, **(F)** REUS replicas visited at least once by individual replicas.

The distribution of distance replicas along the REUS dimension at 310 K is shown in Figures 7A,C,E for Src-PP1, Src-Dasatinib, and Abl-Imatinib, respectively (Supplementary Figure S3A for Src-PP1-rev). All systems show a similar trend, where the population is large at short distances, drops once as the distance increases, and then converges to a constant value. Despite the drop in population, owing to the intensive tuning of the replica parameters, all regions were sampled to an acceptable extent, maintaining a constant overlap and good acceptance ratios between adjacent replicas. For example, in the case of Src-Dasatinib, the initial lack of population in the region of protein-ligand distances 4–8 Å was gradually filled with increasing sampling time, and converged at 250 ns (Supplementary Figures S4A,C,E,G). The lack of population is much less significant at higher temperature replicas (Supplementary Figure S5), indicating that two-dimensional replica exchanges improve the sampling at 310 K.

Figures 7B,D,F (Supplementary Figure S3B) demonstrate the random walks along the REUS dimension. Each of the 240 replicas visited all REUS distances almost perfectly for Src-PP1 and moderately for Src-Dasatinib. In contrast, for Abl-Imatinib, random walks in the vicinity of each region are rather good but the overall random walks are not as efficient, namely, replicas which started at small distances could not reach far distances and vice versa (Figure 7F). This suggests that a large and flexible ligand can be trapped in the vicinity of its starting configuration due to either specific or non-specific interactions with the protein.

#### 3.5.3 Finding the X-Ray Bound Pose in gREST/REUS Simulations

**FIGURE 8:**
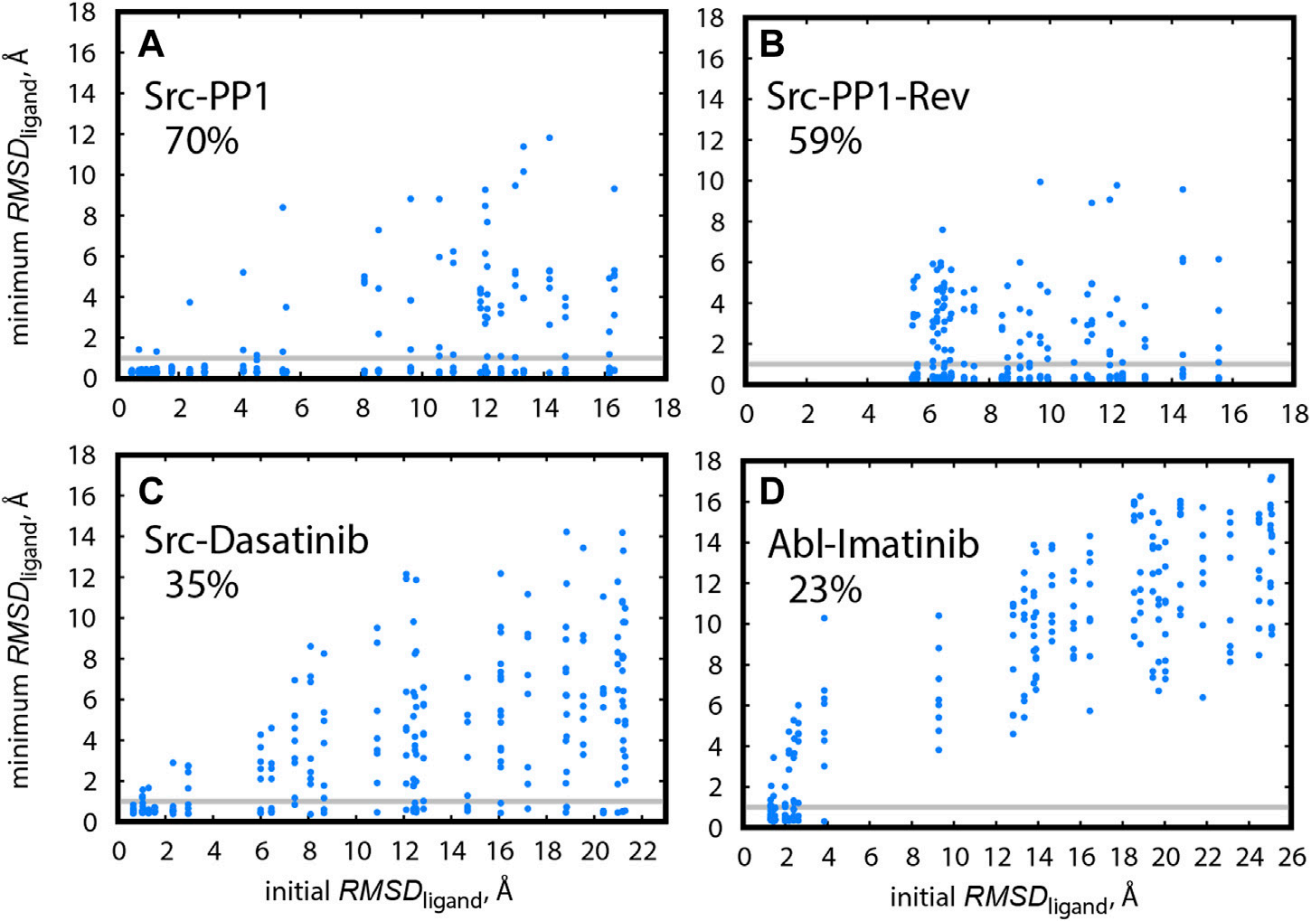
Minimum *RMSD*_ligand_ for replicas during the simulation, plotted against their initial *RMSD*_ligand_, in **(A)** Src-PP1, **(B)** Src-PP1-Rev, **(C)** Src-Dasatinib, and **(D)** Abl-Imatinib. Grey horizontal lines mark *RMSD*_ligand_ = 1 Å. The percentage of replicas that reached the bound pose is written for each system.

Finally, we compared the efficiencies of finding the X-ray bound pose for Src-PP1, Src-Dasatinib, and Abl-Imatinib. Figure 8 shows the minimum *RMSD*_ligand_ for individual replicas as a function of the initial *RMSD*_ligand_ for all simulated systems. We define that a replica reached the bound pose if it had a *RMSD*_ligand_ < 1 Å at least once during the simulation. The hit ratios along the sampling time are also summarized in Supplementary Table S2. For Src-PP1, 70% of the replicas, including those starting from far distances (large initial *RMSD*_ligand_), found the X-ray bound pose (Figure 8A). Notably, the hit ratio was slightly low (59%) in the reverse pulling simulation (Src-PP1-Rev). We find that the initial *RMSD*_ligand_ values are larger than 5 Å, indicating that the Src-PP1-Rev simulation did not include the bound pose, which is nearly identical to the X-ray crystal structure, in its initial structures. Nevertheless, many replicas starting from large *RMSD*_ligand_ values found the bound pose within 500 ns simulations, demonstrating that the gREST/REUS method can efficiently find an unknown bound pose.

The hit ratio drops to 35% for Src-Dasatinib (Figure 8C). However, a fraction of replicas with an initial *RMSD*_ligand_ of ∼6 Å and above still finds the bound pose. For Abl-Imatinib (Figure 8D), which is the most challenging case, the hit ratio was only 23% even though its simulation time (1,000 ns) was the longest among the three systems. There is a gap in *RMSD*_ligand_ values between ∼4 Å and ∼9 Å. Unlike the case of Src-Dasatinib, the replicas above ∼9 Å cannot even reach the vicinity of the binding site (Figure 8D). Therefore, the hit ratio stays around 20% after 250 ns and until 1,000 ns (Supplementary Table S2). These results suggest that Imatinib binding is a considerably rare event and that Imatinib can be trapped at various locations in the vicinity of the binding region before fully entering deep inside the binding pocket. Supplementary Movies S1–S3 show binding events for a single replica for Src-PP1, Src-Dasatinib, and Abl-Imatinib, respectively, and demonstrate the difference in the efficiency of finding the bound pose. Whereas for Src-PP1 the ligand binds and unbinds several times during 500 ns, for Src-Dasatinib, a single binding event of a replica that started from a far distance is observed after ∼550 ns, and for Abl-Imatinib, a replica that started from an intermediate distance binds after ∼250 ns and does not leave the binding site during the rest of the simulation time.

Here we followed the definition of Re et al. (2019) for hitting the bound pose, who deliberately set a strict cutoff of *RMSD*_ligand_ < 1Å. We could set the cutoff slightly larger (for example 1.5 Å) to consider ligand fluctuations around the bound pose. In this case, we obtain hit ratios of 75, 65, 38, and 27% for Src-PP1, Src-PP1-rev, Src-Dasatinib, and Abl-Imatinib, respectively.

## 4 Discussion and Conclusion

In this work, we described a step-by-step procedure for obtaining the optimal parameter settings for efficient gREST/REUS simulations of protein-ligand binding. The protocol, which was demonstrated here for three kinase-inhibitor systems, was validated through an extensive analysis of sampling efficiency based on a total of 660 μs of simulation time and can be applied to protein-ligand systems in general. We demonstrated that while the determination of gREST parameters is rather straightforward and nearly automatic, a particular care is needed in the determination of REUS parameters. First, a proper definition of the protein-ligand distance as the REUS CV, and second, careful tuning of replica space and force constants. Both of these practices can enhance the sampling efficiency. Taking care of these points, gREST/REUS simulations can sample binding events with high statistical accuracy and the obtained trajectories can be used to characterize binding poses and pathways on the free-energy landscape.

The use of protein-ligand distance as CV is a common practice for simulating binding events. Typically, the distance is determined using the COMs of the binding site and the ligand. For flexible ligands with molecular weight of few hundreds, as in the case of Imatinib, the determination of the CV significantly affects replica exchanges in REUS dimension. A lesson from this work is that each COM of the binding site and the ligand should be determined using multiple anchor sites for taking the flexibilities and orientation into account. This is because the flexible ligand can interact with the protein in different conformations and at different parts of the molecule. Even with a proper definition of the protein-ligand distance and well-tunned REUS parameters (replica spacing and force constants), the realization of constant acceptance ratios throughout the REUS dimension is quite dificult as shown for Imatinib. Here, we must add that applying too stiff umbrella potentials during the pulling simulations for obtaining the initial REUS replicas or during the REUS simulation may affect the obtained binding pathways. Thus, we must find the right balance of parameters that will not excessively bias the simulation but will still result in efficient sampling.

We showed that gREST/REUS can fill this gap with the aid of solute temperature exchange. Our results justify performing exchanges in two dimensions while using non-negligible computational resources. Using this protocol, protein-ligand binding simulations, in particular ligands or inhibitors of small or medium sizes, would be successfully performed on massively parallel supercomputers or GPU clusters.

Although good random walks in the replica space were observed in all three cases, simulation results of Abl-Imatinib suggest that efficient conformational sampling of Imatinib around the binding site of c-Abl kinase is still challenging. Unlike for Src-PP1 and Src-Dasatinib simulations, we could not observe many binding/unbinding events for Imatinib, especially of replicas initiated from far distances. Observing efficient random walks along the whole REUS range is important for visualizing the binding pathway. We learned that the problem is harder as the ligand size increased from PP1 (easy) to Dasatinib (moderate) to Imatinib (difficult), especially for obtaining the whole binding pathway. To further enhance the sampling for flexible ligands, consideration of a CV other than protein-ligand distance or an extension of the current scheme would be necessary. Considering the very slow unbinding rate of Imatinib, more drastic acceleration, such as simulations at higher solvent temperatures or enhancement of the c-Abl kinase domain motions might be introduced in the gREST/REUS simulations.

Another practical drawback of the gREST/REUS ligand-binding simulations is that huge computational resources are required for them. In this study, we used 240 replicas in the 2D-REMD for each of the three cases. Without the use of Fugaku or other massively parallel supercomputers, it is not easy to access such huge resources. One way to overcome the problem is to replace gREST or REUS with other enhanced sampling methods with less computational costs. We previously developed GaREUS (Gaussian accelerated replica-exchange umbrella sampling) (Oshima et al., 2019) by replacing gREST in gREST/REUS into GaMD (Miao and McCammon, 2017). We were able to significantly reduce the number of replicas using GaREUS while keeping the sampling strategy and efficiency, because GaREUS requires the same number of replicas as 1D-REUS. The use of such low-cost enhanced sampling methods is necessary for investigating molecular mechanisms for many other kinase-inhibitor binding processes.

## Data Availability

The original contributions presented in the study are included in the article/Supplementary Material, further inquiries can be directed to the corresponding author.

## Footnotes

1
https://www.r-ccs.riken.jp/en/fugaku/.
