# Supplementary material for: Practical Protocols for Efficient Sampling of Kinase-Inhibitor Binding Pathways Using Two-Dimensional Replica-Exchange Molecular Dynamics
Source: Front Mol Biosci. 2022 Apr 29;9:878830. doi: 10.3389/fmolb.2022.878830 (PMC9099257; doi:10.3389/fmolb.2022.878830)
Supplement: Supplementary file 4 [file DataSheet1.docx]

***Supplementary Material***

**Supplementary Text**

Description of the gREST/REUS method.

**Supplementary Figures**

Figure S1. Scheme describing the gREST/REUS method.

Figure S2. Efficiency of sampling in the gREST dimension for various systems.

Figure S3. Efficiency of sampling in the REUS dimension for simulation of Src-PP1-Rev.

Figure S4. Efficiency of sampling in the REUS dimension for Src-Dasatinib at 310 K at different times during the simulation.

Figure S5. Efficiency of sampling in the REUS dimension for Src-Dasatinib at different solute temperatures.

**Supplementary Tables**

Table S1. gREST/REUS simulation parameters.

Table S2. The percentage of replicas that reached the bound pose at increasing times during the simulation.

**Supplementary Movies**

Movie S1. Trajectory of Src-PP1 simulation (replica 214, initial protein-ligand distance was 4.3 Å). Protein is shown in cartoon representation. Ligand is shown as balls.

Movie S2. Trajectory of Src-Dasatinib simulation (replica 150, initial protein-ligand distance was 23.1 Å). Protein is shown in cartoon representation. Ligand is shown as balls.

Movie S3. Trajectory of Abl-Imatinib simulation (replica 159, initial protein-ligand distance was 8.3 Å). Protein is shown in cartoon representation. Ligand is shown as balls.

**The two-dimensional gREST/REUS method**

In the two-dimensional gREST/REUS method, *n*×*m* initial replicas are prepared where *n* is the number of replicas in the gREST dimension (i.e. solute temperature) and *m* is the number of replicas in the REUS dimension (in the current case protein-ligand distance). Each initial replica carries a unique solute temperature and a unique protein-ligand distance. During the simulation, exchange attempts are performed alternatively in the gREST and the REUS dimensions (Figure S1). By performing exchanges in both dimensions, we obtain the benefits of both the gREST and the REUS methods, which further enhances the sampling.

| 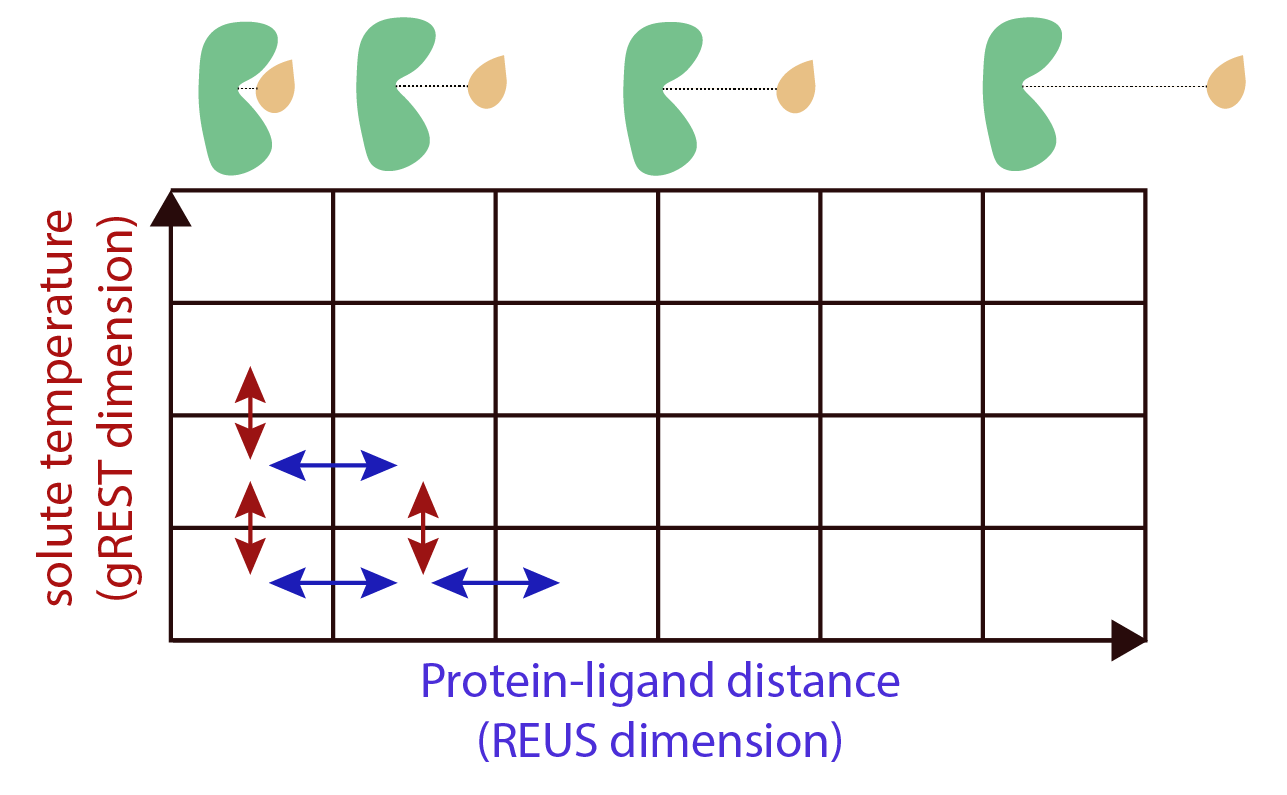 |
| --- |
| **Figure S1.** Scheme detailing the exchange patterns in the gREST/REUS method. |

| 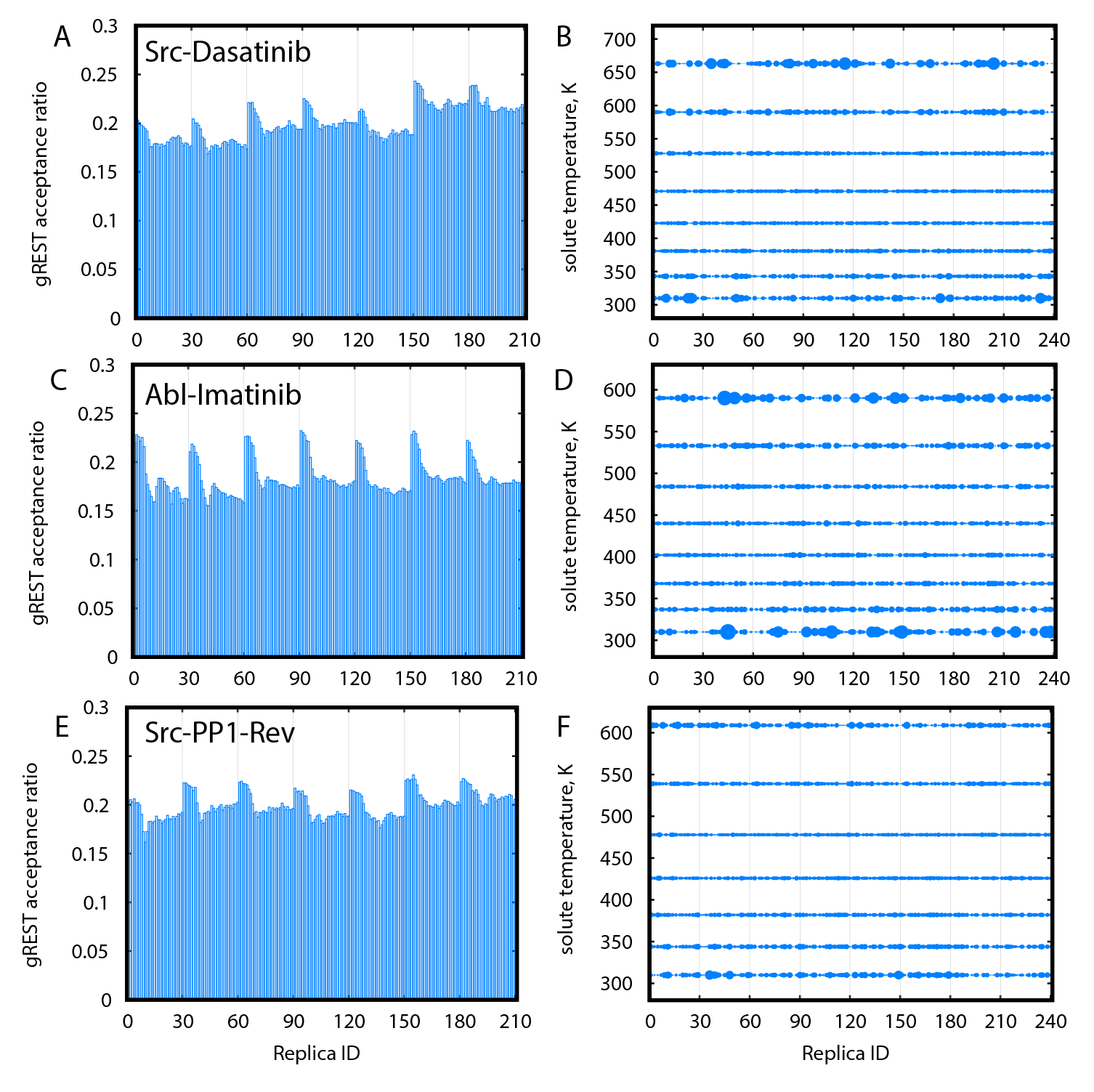 |
| --- |
| **Figure S2.** Sampling in gREST dimension for gREST/REUS simulations of Src‑Dasatinib (A, B, 750 ns), Abl-Imatinib (C, D, 1,000 ns), and Src-PP1-Rev (E, F, 500 ns). Left column: Acceptance ratios between each replica and the gREST replica adjacent and above it. Right column: Relative population for each replica at different solute temperatures. Sphere size is proportional to the population. Replicas assigned different initial solute temperatures are separated by vertical lines, where replicas 1–30 were assigned the initial temperature of 310 K (T1), replicas 31–60 were assigned the initial temperature of T2, etc. |

| 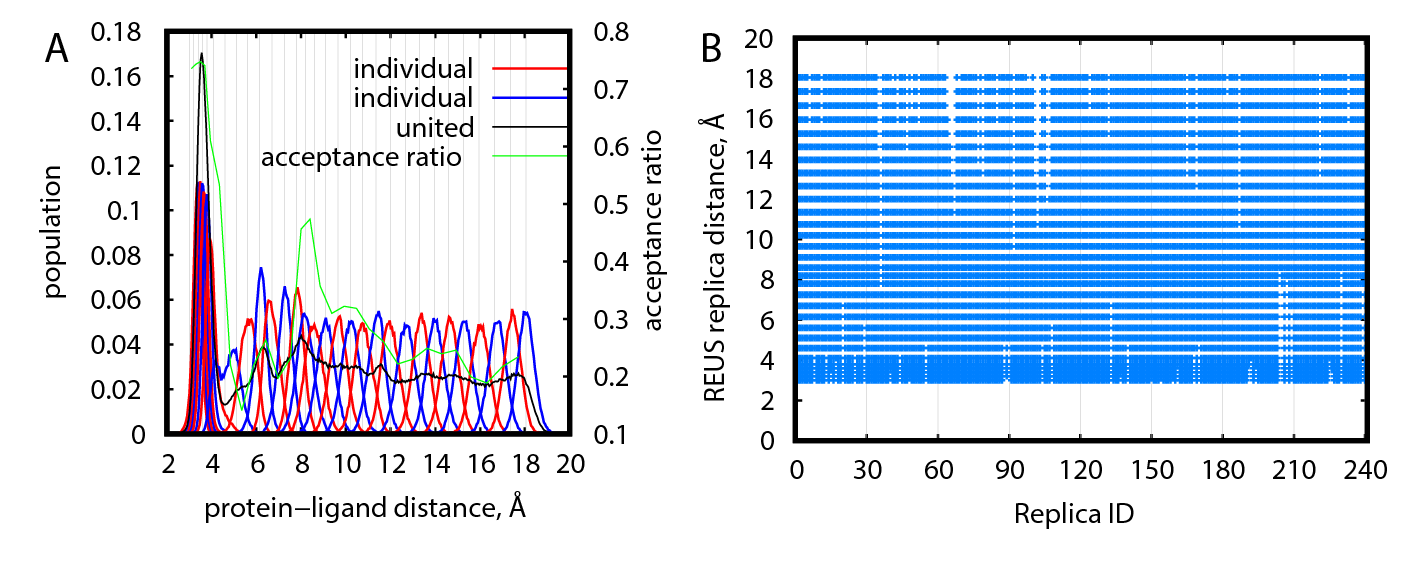 |
| --- |
| **Figure S3.** Efficiency of sampling in REUS space for gREST/REUS simulations at 310 K for Src-PP1-Rev. (A) Distribution of replicas according to their REUS distance. Distributions of adjacent individual replicas (“individual”) are shown in alternating red/blue lines for better visibility. Distributions of all replicas (“united”) are shown in black lines. Population values for “united” data were scaled to match the “individual” populations. Acceptance ratios between adjacent REUS replicas are shown in green lines. (B) REUS replicas visited at least once by individual replicas. |
| 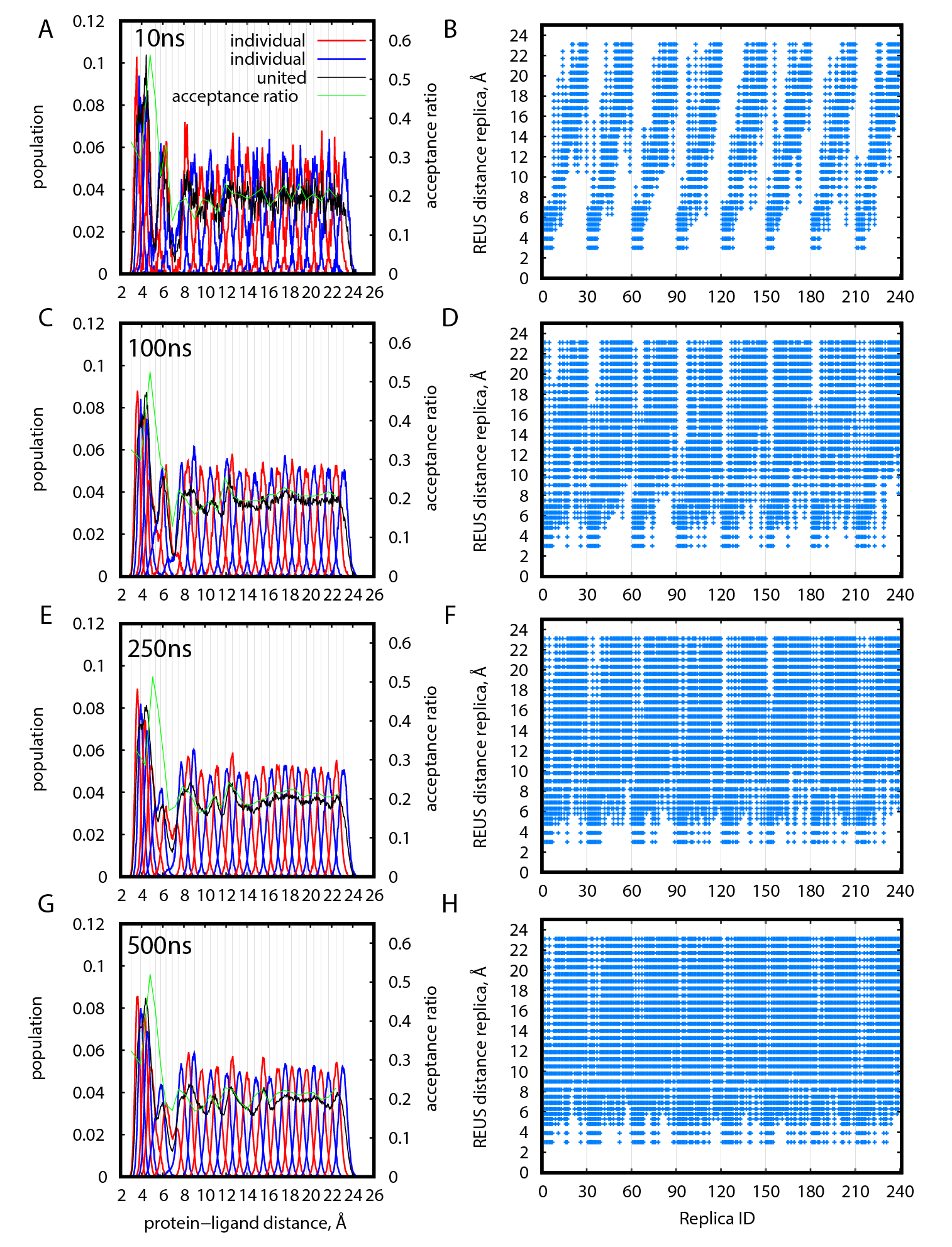 |
| **Figure S4.** Efficiency of sampling in REUS space for gREST/REUS simulations at 310 K of Src-Dasatinib. (A), (C), (E), (G): Distribution of replicas according to their REUS distance at increasing times. Distributions of adjacent individual replicas (“individual”) are shown in alternating red/blue lines for better visibility. Distributions of all replicas (“united”) are shown in black lines. Population values for “united” data were scaled to match the “individual” populations. Acceptance ratios between adjacent REUS replicas are shown in green lines. (B), (D), (F), (H): REUS distances visited at least once by individual replicas at increasing times. |

| 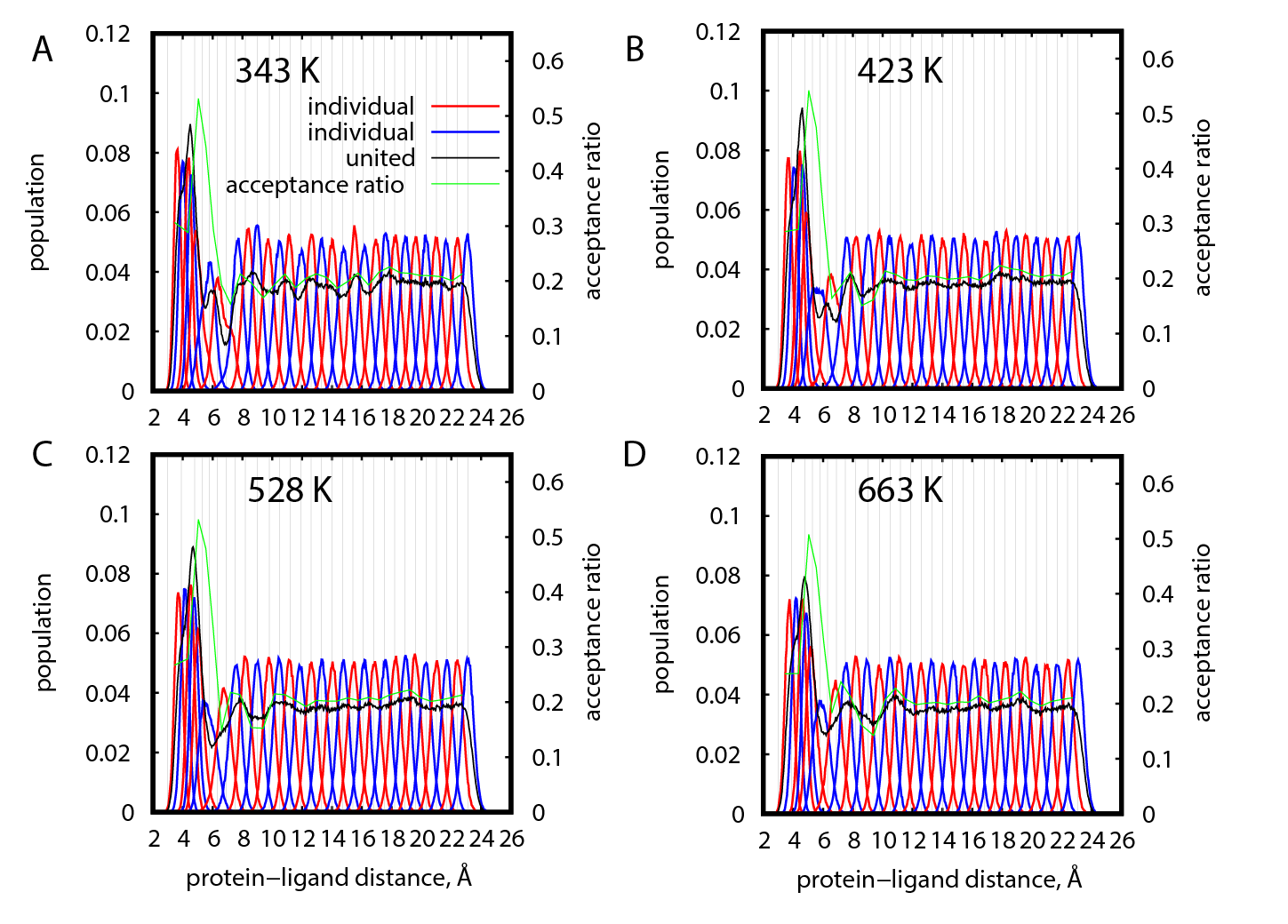 |
| --- |
| **Figure S5.** Distribution of replicas according to their REUS distance for gREST/REUS sampling simulations at different gREST temperatures (T2, T4, T6, and T8 for A, B, C, and D, respectively) for Src-Dasatinib after 750 ns. Distributions of adjacent individual replicas (“individual”) are shown in alternating red/blue lines for better visibility. Distributions of all replicas (“united”) are shown in black lines. Population values for “united” data were scaled to match the “individual” populations. Acceptance ratios between adjacent REUS replicas are shown in green lines. |

| **Table S1. The gREST/REUS simulation parameters** | | | | | |
| --- | --- | --- | --- | --- | --- |
| System | Solute temperatures, K | gREST solute region residues^1^ | REUS replica distance, Å | REUS replicas force constants, kcal/mol/Å^2^ |  |
| Src-PP1 | 310, 344, 382, 426, 478, 539, 609, 692 | 15, 23, 35, 37, 78, 80, 81, 83, 85, 87, 135, 146 | 3.0, 3.6, 4.0, 4.3, 4.6, 4.9, 5.2, 5.5, 5.8, 6.1, 6.6, 7.1, 7.7, 8.3, 8.9, 9.5, 10.1, 10.7, 11.3, 11.9, 12.5, 13.1, 13.7, 14.3, 14.9, 15.5, 16.1, 16.7, 17.3, 17.9 | 2.0, 2.0, 4.0, 4.0, 4.0, 4.0, 4.0, 3.0, 2.0, 2.0, 2.0, 2.0, 2.0, 2.0, 2.0, 2.0, 2.0, 2.0, 2.0, 2.0, 2.0, 2.0, 2.0, 2.0, 2.0, 2.0, 2.0, 2.0, 2.0, 2.0 |  |
| Src-PP1-Rev | 310, 344, 382, 426, 478, 539, 609, 692 | 15, 23, 35, 37, 78, 80, 81, 83, 85, 87, 135, 146 | 3.0, 3.2, 3.4, 3.6, 3.8, 4.1, 4.6, 5.1, 5.6, 6.15, 6.7, 7.25, 7.8, 8.2, 8.6, 9.1, 9.65, 10.2, 10.75, 11.35, 12.0, 12.65, 13.3, 13.95, 14.6, 15.25, 15.95, 16.65, 17.35, 18.05 | 4.0, 4.0, 4.0, 4.0, 4.0, 4.0, 3.0, 3.0, 3.0, 3.0, 3.0, 3.0, 3.0, 2.0, 2.0, 2.0, 2.0, 2.0, 2.0, 2.0, 2.0, 2.0, 2.0, 2.0, 2.0, 2.0, 2.0, 2.0, 2.0, 2.0 |  |
| Src-Dasatinib | 310, 343, 381, 423, 471, 528, 590, 663 | 15, 23, 35, 37^2^, 78, 80, 81, 83, 85^2^, 87, 135, 146 | 3.00, 3.90, 4.80, 5.30, 5.80, 6.30, 6.90, 7.50, 8.20, 9.00, 9.80, 10.50, 11.20, 11.90, 12.60, 13.30, 14.00, 14.70, 15.40, 16.10, 16.80, 17.50, 18.20, 18.90, 19.60, 20.30, 21.00, 21.70, 22.40, 23.10 | 2.0, 2.0, 2.0, 2.0, 2.0, 2.0, 2.0, 2.0, 2.0, 2.0, 2.0, 2.0, 2.0, 2.0, 2.0, 2.0, 2.0, 2.0, 2.0, 2.0, 2.0, 2.0, 2.0, 2.0, 2.0, 2.0, 2.0, 2.0, 2.0, 2.0 |  |
| Abl-Imatinib | 310, 337, 368, 402, 440, 484, 533, 590 | 25, 30, 46, 48, 63, 67, 76, 90, 92, 94, 95, 137, 139, 140^2^, 157, 158, 159 | 2.7, 3.4, 4.1, 4.8, 5.5, 6.2, 6.9, 7.6, 8.3, 9.0, 9.7, 10.4, 11.1, 11.8, 12.5, 13.2, 13.9, 14.6, 15.3, 16.0, 16.7, 17.4, 18.1, 18.8, 19.5, 20.2, 20.9, 21.6, 22.3, 23.0 | 2.0, 2.0, 2.0, 2.0, 2.0, 2.0, 2.0, 2.0, 2.0, 2.0, 2.0, 2.0, 2.0, 2.0, 2.0, 2.0, 2.0, 2.0, 2.0, 2.0, 2.0, 2.0, 2.0, 2.0, 2.0, 2.0, 2.0, 2.0, 2.0, 2.0 |  |
| ^1^Solute region also includes the ligand  ^2^Residue added to obtain a neutral solute region | | | | | |

| **Table S2. The percentage of replicas that reached the bound pose at increasing times during the simulation** | | | | | | | |
| --- | --- | --- | --- | --- | --- | --- | --- |
| System | 10 ns | 50 ns | 100 ns | 250 ns | 500 ns | 750 ns | 1000 ns |
| Src-PP1 | 39 | 48 | 55 | 63 | 70 | # | # |
| Src-PP1-Rev | 8 | 23 | 32 | 47 | 59 | # | # |
| Src-Dasatinib | 18 | 22 | 23 | 28 | 33 | 35 | # |
| Abl-Imatinib | 13 | 19 | 20 | 22 | 23 | 23 | 23 |
